# A Children's Nurse's View of Registration

**Published:** 1919-02-15

**Authors:** M. P. Ferrier


### A Children's Nurse's View of Registration.

*To the Editor of* THE HOSPITAL.

SIR,—I was at the meeting on January 23 at the Royal Society of Medicine which took place for the College of Nursing. In regard to the question raised by Sir Cooper Perry, referring to children's hospitals, I am anxious to put forward a suggestion about the same. The nurses of these hospitals have three years' training, yet they are not eligible for the College Register. Surely this certificate could be augmented by one year's training in a general hospital, doing six months in the wards for women and six months in the wards for men. I feel sure that any nurse who had trained for three years with children, with the extra year's training, would be equal to a three-years' general trained nurse. The knowledge gained in the wards of a children's hospital entails, in my opinion, great patience, tact, and experience, for a so trained nurse to have to go through her three years general training is a tax on her strength and reserve forces which is unfair to expect.

During my own training I remember at the least six nurses who had already spent three years in a children's hospital, and yet they had to begin again at the bottom. I myself, since my general training, have had much experience with children amongst other nursing experiences, so write feelingly regarding the nurses in children's hospitals.

This is a question that could be easily arranged by the General Hospital Training Schools and the Children's Hospital. It is only a question of affiliating the latter with the former, and before the nurse receives her certificate she can gain her one year's experience in a general hospital. This form of affiliation is already done with special and general hospitals, therefore why not with the children's hospitals? I am sure there would be no difficulty in staffing these hospitals. Such a plan would encourage those women who prefer children's hospitals, and an extra year's senior training in a general hospital would enable them to take their proper standing with a three-years' general trained nurse.

I am a recently joined member of the College of Nursing, and hope something will be done to enable the nurses of children's hospitals to take their place on the register of the College of Nursing. M. P. FERRIER.

